# Motor symptoms of Parkinson's disease are affected by temperature: A controlled pilot study

**DOI:** 10.1002/brb3.3369

**Published:** 2024-01-06

**Authors:** Ting Huang, Xi‐Xi Wang, Chen‐Yu Gao, Jin‐Ying Zhao, Rong‐Rong Pan, Hui‐Hui Jin, You‐Yong Tian, Ying‐Dong Zhang

**Affiliations:** ^1^ Department of Neurology, Nanjing First Hospital Nanjing Medical University Nanjing China; ^2^ Department of Neurology, Affiliated Zhongda Hospital, School of Medicine, Institute of Neuropsychiatry, Key Laboratory of Developmental Genes and Human Disease Southeast University Nanjing China; ^3^ Department of Mental Health and Public Health, Faculty of Life and Health Sciences, Shenzhen Institute of Advanced Technology Chinese Academy of Sciences Shenzhen China

**Keywords:** hyperthermic baths, Parkinson's disease, physiotherapy, temperature

## Abstract

**Purpose:**

The motor symptoms (MS) of Parkinson's disease (PD) have been affecting the quality of life in patients. In clinical practice, most patients with PD report that MS are more severe in winter than in summer, and hyperthermic baths (HTB) could temporarily improve MS. The study aimed to evaluate the effects of seasonal variation and aquatic thermal environment of HTB on the MS of PD.

**Patients and methods:**

A cross‐sectional study of 203 Chinese Han patients was performed. Univariate and multivariate analyses were performed to analyze seasonal variation in MS relative to baseline data (sex, age at onset, duration, season of birth, Hoehn and Yahr stage, family history, levodopa equivalent dose, and the effect of HTB on MS). Ten subjects participated in the HTB study, and one patient dropped out. The paired Wilcoxon rank test was used to assess the differences in the Movement Disorder Society‐United Parkinson's disease Rating Scale (MDS‐UPDRS) part III motor examination total scores and the modified Webster Symptoms Score between non‐HTB and before HTB and between non‐HTB and after HTB.

**Results:**

The improvement of MS after HTB was an independent risk factor for seasonal variation in MS (OR, 25.203; 95% CI, 10.951–58.006; *p* = .000). Patients with PD had significant improvements in the MDS‐UPDRS part III motor examination total scores, especially in bradykinesia (*p* = .043), rigidity (*p* = .008), posture (*p* = .038), and rest tremor amplitude (*p* = .047).

**Conclusion:**

Seasonal variation in temperature and water temperature of HTB may affect MS in some patients with PD. Simple HTB could be recommended as physiotherapy for patients with PD who report temperature‐sensitive MS.

## Graphical abstract

In order to evaluate the effects of seasonal variation and aquatic thermal environment of hyperthermic baths (HTB) on the motor symptoms (MS) of Parkinson's disease (PD). This study assessed the differences in the Movement Disorder Society‐United Parkinson's disease Rating Scale (MDS‐UPDRS) part III motor examination total scores between non‐HTB and before HTB and between non‐HTB and after HTB. We found that seasonal variation in temperature and water temperature of HTB may affect MS in some PD patients. Simple HTB could be recommended as physiotherapy to PD patients who report temperature‐sensitive MS.

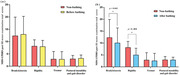

## 1 INTRODUCTION

Parkinson's disease (PD) is the second most common progressive neurodegenerative disorder and is clinically characterized by motor symptoms (MS), such as bradykinesia, resting tremor, rigidity, and postural instability. The pathological hallmark of PD is the loss of dopaminergic neurons in the substantia nigra (Kalia & Lang, 2015; Opara et al., 2017). In clinical practice, most patients with PD complain that the MS of PD in winter (low temperature, less sunlight) are more severe than in summer. Coldness of the lower limbs of PD usually occurs in winter and is often accompanied by pain, potentially causing difficulty in walking or standing (Kataoka & Ueno, 2016). It is also reported that hypothermic patients with PD may present with worsening bradykinesia and rigidity (Li & Lou, 2012). An ecological study suggested that temperature is associated with a seasonal fluctuation in the levodopa equivalent dose (LED), increasing by 4.2% in January and decreasing by 4.5% in July (Rowell et al., 2017). However, A study on 546 patients with PD reported no seasonal fluctuation in MS severity when measured with the Unified Parkinson's Disease Rating Scale (UPDRS) (Postuma et al., 2005). A cross‐sectional retrospective study on 372 consecutive patients with PD reported that nonmotor symptoms of PD fluctuated throughout the year and were more severe in winter than in summer (van Wamelen et al., 2019). Despite some efforts to explore the possibility, there is not yet clear evidence for seasonal fluctuations in the MS of PD. Therefore, we performed a cross‐sectional study to further evaluate seasonal variation in MS of patients with PD.

In clinical practice, most of the patients with PD complain that MS are more severe in winter, and these patients also feel an improvement in MS after hyperthermic baths (HTB). HTB are one of the classical salus per aquam (spa) treatments and have thermal, mechanical, and chemical effects on health (An et al., 2019; Bender et al., 2005; Fioravanti et al., 2011; Sukenik et al., 1999). Some studies have shown that the thermal stimulation produced by HTB may influence muscle tone and pain intensity, helping to reduce muscle spasm, increase the pain threshold in nerve endings (Fioravanti et al., 2011), and improve the range of motion of joints by increasing the extensibility of collagen‐rich tissues, such as tendons, fasciae, and articular capsules (Gomes et al., 2019; Petrofsky et al., 2013; Sukenik et al., 1999). As a form of physiotherapy, HTB have been proven to be effective in improving dynamic balance and gait speed in different neurological diseases, such as stroke (Montagna et al., 2014), peripheral neuropathies (Zivi et al., 2018), and multiple sclerosis (Marinho‐Buzelli et al., 2015; Methajarunon et al., 2016). Aquatic physiotherapy combines an aquatic thermal environment with strategy training, cycling, tai chi, progressive resistance strength training, walking programs, and therapeutic dancing, which is also used in exercises for people with early PD. Compared to studies with patients who underwent therapy on dry land, studies based on aquatic physiotherapy have gradually confirmed the improvements in gait and postural stability in patients with PD (Masiero et al., 2019; Palamara et al., 2017; Perez de la Cruz, 2017; Vivas et al., 2011; Volpe et al., 2014). However, reports using simple HTB for patients with PD are scarce.

Therefore, to further evaluate the effects of seasonal variation and the aquatic thermal environment of HTB on the MS of PD, we conducted a two‐phase study to analyze the possible association between temperature and MS.

## 2 METHODS

### 2.1 Participants

Phase 1 of the study was carried out at the Nanjing First Hospital and Nanjing Brain Hospital during a period from November 2019 to August 2022. A total of 203 consecutive Chinese Han patients with a diagnosis of idiopathic Parkinson's disease (IPD) were recruited from the outpatient clinic. These patients with PD came from Nanjing and its surrounding areas, which are located in the mid‐latitude zone of the east coast of the Asian continent. The region belongs to the East Asian monsoon climate zone, which has mild climatic characteristics with four distinct seasons and hot summers and cold winters. The average temperature is 3.0°C in winter and 25.9°C in summer.

Phase 2 of the study was carried out from September 2022 to October 2022. Based on the clinical baseline data from Phase 1, a total of 10 subjects participated in the HTB study, and one dropped out for personal reasons during the study. The inclusion criteria for subjects were as follows: (1) ≥18 years of age, (2) diagnosis of IPD based on the Movement Disorder Society clinical diagnostic criteria for PD, (3) subjective feeling that MS of PD were affected by seasonal variation (i.e., MS were more severe in winter than in summer) and improved after HTB, and (4) no change in the patient's PD medication regimen during the study. The exclusion criteria included (1) atypical or secondary parkinsonism, (2) other neurologic illness or injury (traumatic brain injury, ischemic/hemorrhagic stroke, Alzheimer's disease, and epilepsy), (3) previous cardiovascular disease (symptomatic bradycardia, severe postural hypotension, symptomatic coronary insufficiency, and severe organic heart damage), (4) unstable psychiatric disorders such as schizophrenia or major depression, (5) acute or chronic inflammatory diseases, (6) osteoarticular diseases which may be affected by temperature (osteoarthritis and rheumatoid arthritis), (7) dementia (Mini‐Mental State Examination [MMSE] < 24 and Montreal Cognitive Assessment [MOCA] < 26), and (8) failure to cooperate with the study requirements and poor compliance. The study was approved by the ethics committee of Nanjing First Hospital (No. KY20190926‐05). The study was conducted strictly in accordance with the implementation rules of the Regulations on the Administration of Medical Institutions issued by the State Council of the People's Republic of China. All participants gave written informed consent.

### 2.2 Study design

**FIGURE 1:**
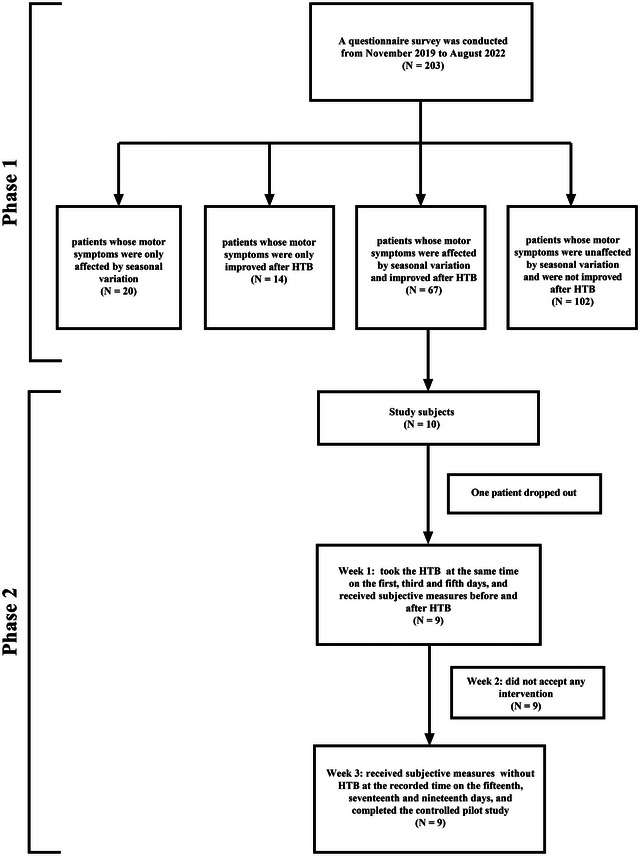
Flow program of the study. HTB, hyperthermic baths.

To evaluate the relationship between temperature and MS, our study was divided into two phases, as described below (Figure 1).

### 2.3 Phase 1: A cross‐sectional study

The baseline data for the patients with PD were collected and analyzed, including sex, age at onset, PD duration, the season of birth, Hoehn and Yahr (H&Y) stage, family history, levodopa equivalent dose (LED), the effect of HTB on MS, and the effect of seasonal variation on MS.

A survey regarding subjective feelings about MS of PD was conducted with a questionnaire to assess the association between the effect of HTB on MS and the effect of seasonal variation on MS. The questionnaire comprised four questions associated with two factors, which included the effect of seasonal variation on MS and the effect of HTB on MS. The four questions are as follows:

1. Do you feel that the MS of PD are affected by seasonal variation?
2. What do you think the differences are in the MS of PD between winter (low temperature, less sunshine) and summer?
3. Do you feel that the MS of PD are affected by HTB?
4. Do you feel that the MS of PD are improved after HTB?

Each question was answered according to a three‐category scale (0–2). For questions 1 and 3, a score of 0 corresponded to no influence. A score of 1 meant a possible influence, and a score of 2 meant a positive influence. For question 2, a score of 0 meant no difference, a score of 1 meant that MS were more severe in summer, and a score of 2 meant that MS were more severe in winter. For question 4, a score of 0 meant no improvement, a score of 1 meant a possible improvement, and a score of 2 meant a certain improvement (Table S1). A score of 2 for both questions 1 and 2 indicated that the MS of PD were affected by seasonal variation (i.e., MS were more severe in winter than in summer). A score of 2 for both questions 3 and 4 indicated that the MS of PD were improved after HTB.

### 2.4 Phase 2: The HTB study

The objective of the HTB study was to evaluate whether HTB could improve MS in PD. Based on clinical baseline data from phase 1, subjects who met the inclusion criteria underwent a complete history and physical examination, including an assessment using the Movement Disorder Society‐United Parkinson's Disease Rating Scale (MDS‐UPDRS), H&Y stage, MMSE, and MOCA. Each subject received two subjective measures (the MDS‐UPDRS part III motor examination total scores and the modified Webster Symptoms Score) nine times in an open medication state (open state). Each subject took HTB in an open medication state at the same time on the first, third, and fifth day. In the first week, they received subjective measures before and after the HTB. The time at which the subjective measures began after the HTB was recorded. In the second week, no subjects accepted any intervention. To avoid the effects of fluctuations in dopaminergic drug concentrations, subjective measures were given without HTB at the recorded times on the 15th, 17th, and 19th day of the third week.

**FIGURE 2:**
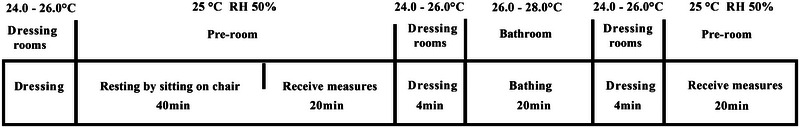
Time schedule of the experiment. RH, relative humidity.

To avoid the influence of seasonal temperature, the mean temperatures of the dressing rooms and bathrooms were controlled at 24.0–26.0°C and 26.0–28.0°C, respectively. We used initial water temperatures of 41 and 40°C, which declined by 1.0–1.5°C after 10 min of bathing. Although the water temperature could not be controlled automatically, it was strictly controlled between 39 and 41°C during bathing, as these are the most comfortable temperatures for baths and showers (Hong et al., 1991; Kamata et al., 1992). First, subjects put on long‐sleeved shirts and trousers and sat in the pre‐room, which had an air temperature of 25°C and a relative humidity of 50% for 1 h. Then, their clothes were removed within 4 min, and they immersed themselves in the bathtub with their legs extended forward for 20 min, which is the most comfortable bath time for the body. The surface of the water in the bathtub reached the level of the axilla. After the HTB, the subjects wore new dry clothes to receive subjective measures in the pre‐room (Figure 2).

### 2.5 Statistical analysis

Statistical analyses were performed using SPSS version 23.0 (IBM Corp. Software). Two‐sided *p*‐values of < .05 and two‐sided *p*‐values of < .15 were considered significant and trending toward significance, respectively. Descriptive statistics of categorical variables were presented as counts and percentages, while continuous variables were reported as the mean and standard deviation (*M* [±SD]) or median and interquartile range (*M* [P_25_–P_75_]) according to the normality of the distribution (checked using the Shapiro–Wilk test). To compare groups from phase 1 of the study, we used the Mann–Whitney *U* test for nonparametric continuous variables, the Cochran–Armitage trend test for ordered categorical variables, and the chi‐square test for disordered categorical variables. Furthermore, the relationship between the effect of seasonal variation on MS and the variables trending toward significance (*p* < .15) was assessed by binary logistic regression analysis. Finally, the paired Wilcoxon rank test was used to adequately assess the differences in the MDS‐UPDRS part III motor examination total scores and the modified Webster Symptoms Score for nine subjects between non‐HTB and before HTB and between non‐HTB and after HTB.

## 3 RESULTS

### 3.1 Subjects

In phase 1 of the study, a total of 203 outpatients were enrolled. Demographic information and baseline characteristics are illustrated in Table 1. According to the effect of seasonal variation on the patient's MS, 203 patients were divided into two groups: the affected group (patients with a subjective perception that MS were affected by seasonal variation; *n* = 87) and the unaffected group (patients with a subjective perception that MS were unaffected by seasonal variation; *n* = 116). Among them, 20 patients (9.85%) only subjectively felt that MS were affected by seasonal variation, 14 patients (6.90%) only subjectively felt that MS were improved after HTB, 67 patients (33%) subjectively felt that MS were affected by seasonal variation and improved after HTB, and 102 patients (50.25%) subjectively felt that MS were unaffected by seasonal variation and were not improved after HTB.

**TABLE 1:** Univariate analysis of continuous and categorical variables in patients affected and unaffected by seasonal variation.

	Seasonal variation		
	Affected	Unaffected	
Characteristics	(*n* = 87)	(*n* = 116)	*p* value^a^
Males, *n* (%)	55 (63.22)	59 (50.86)	.079^*^
Age at onset (years), *M* (P25–P75)	62 (56.00–68.00)	65 (58.00–71.00)	.034^**^
PD duration (years), *M* (P25–P75)	5 (3.00–10.00)	3 (2.00–6.00)	.000^**^
Season of birth, *n* (%)			
Winter (Dec–Feb)	15 (17.24)	41 (35.34)	.030^**^
Spring (March–May)	19 (21.84)	22 (18.97)	
Summer (June–Aug)	25 (28.74)	29 (25.00)	
Autumn (Sep–Nov)	28 (32.18)	24 (20.69)	
Hoehn and Yahr stag *n* (%)			
I	20 (22.99)	44 (37.93)	.074^*^
II	23 (26.44)	26 (22.41)	
III	30 (34.48)	31 (26.72)	
IV	14 (16.09)	13 (11.21)	
V	0 (0)	2 (1.72)	
Family history, *n* (%)	14 (16.09)	16 (13.79)	.648
LED, *M* (P25–P75)	412.5 (275.00–650.00)	300.00 (200.00–471.88)	.001^**^
Patients with improvement in MS of PD after HTB, *n* (%)	67 (77.01)	14 (12.07)	.000^**^

Abbreviations: HTB, hyperthermic baths; LED, Levodopa equivalent dose; M (P25–P75), median and interquartile range; MS, motor symptoms; PD, Parkinson's disease.

^a^Efficacy analysis: the Mann–Whitney *U* test, chi‐square test, and Cochran–Armitage trend test.

^*^Trending toward significance; ^**^Significant difference.

In phase 2 of the study, a total of 10 outpatients were enrolled. One patient dropped out during the study. Demographic information and baseline characteristics are illustrated in Table S2.

### 3.2 Outcomes

In phase 1 of the study, the univariate analysis revealed that the risk factors for the affected group were age at onset (*p* = .034), PD duration (*p* = .000), season of birth (*p* = .030), LED (*p* = .001), and the improvement of MS after HTB (*p* = .000) (Table 1). There was no significant difference in sex, H&Y stage, and family history between the two groups (*p* > .05; Table 1). However, the binary logistic regression analysis revealed that the improvement of MS after HTB was an independent risk factor for seasonal variation in MS (odds ratio [OR], 25.203; 95% confidence interval [CI], 10.951–58.006; *p* = .000; Table 2). Compared with the MS of unaffected patients, the MS of affected patients were more susceptible to improvement after HTB. Besides, there was a clear trend in LED for seasonal variation in MS (*p* = 0.095; Table 2).

**TABLE 2:** Logistic model analysis for risk factors included in phase 1 of the study.

Risk factor	OR	95% CI	*p* value^a^
males	1.458	0.633–3.357	.376
Age at onset (years)	0.993	0.951−1.038	.764
Symptoms duration (years)	1.063	0.972−1.162	.180
Season of birth			
Winter (Dec–Feb)			.358
Spring (March–May)	1.903	0.575–6.301	.292
Summer (June–Aug)	1.209	0.391–3.736	.741
Autumn (Sep–Nov)	2.433	0.819–7.234	.110^*^
Hoehn and Yahr stage			
I			.968
II	0.971	0.316–2.979	.958
III	0.678	0.219–2.096	.500
IV	0.741	0.178–3.083	.681
V	0.000		.999
LED	1.002	1.000–1.004	.095^*^
Patients with improvement in MS of PD after HTB	25.203	10.951–58.006	.000^**^

Abbreviations: 95% CI, 95% confidence interval; LED, Levodopa equivalent dose; OR, odds ratio.

^a^Efficacy analysis: the binary logistic regression analysis.

*Trending toward significance; **Significant difference.

**FIGURE 3:**
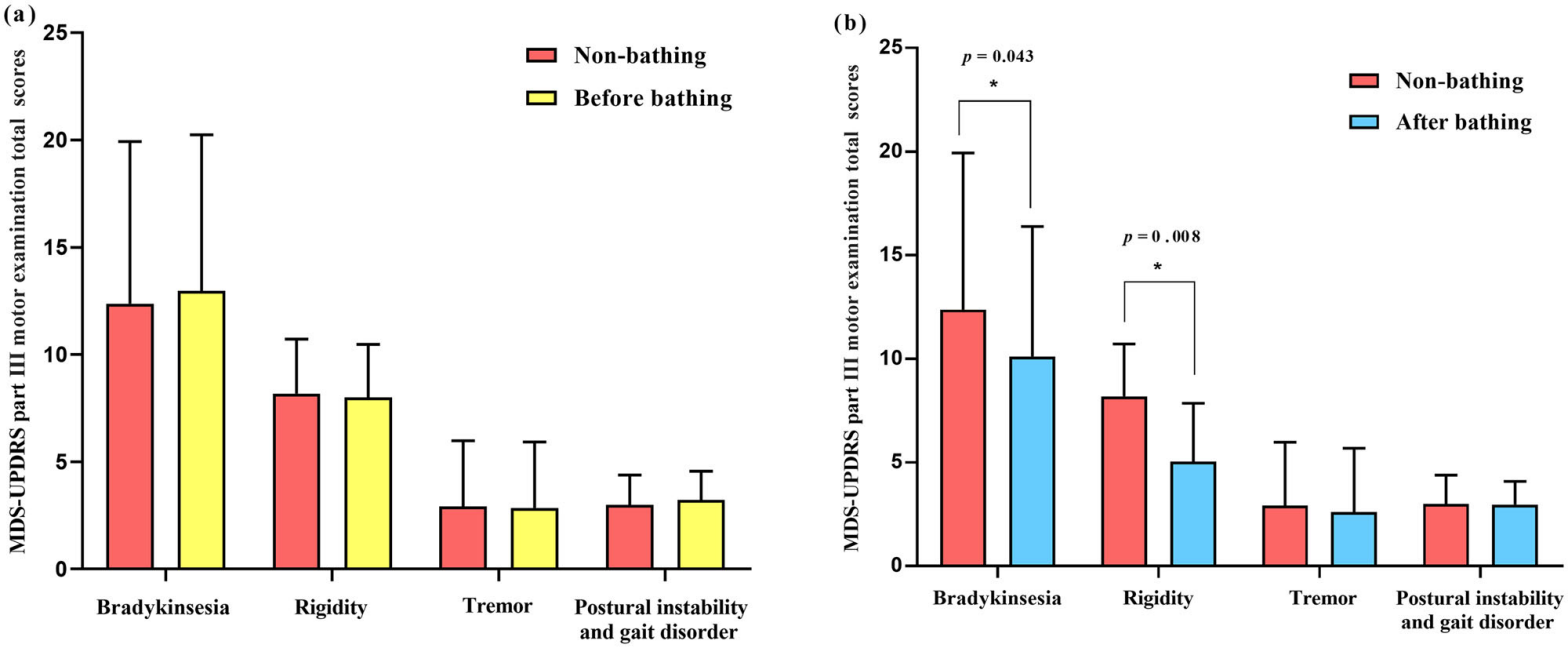
Symptoms of bradykinesia and rigidity could be improved after hyperthermic baths (HTB). (a) The improvement of motor symptoms between non‐HTB and before HTB and (b) the improvement of motor symptoms between non‐HTB and after HTB. *Significant difference (*p* < .05).

In phase 2 of the study, the MDS‐UPDRS part III motor examination total scores of non‐HTB before HTB and after HTB were 24.33 (22.33–30.83), 24.33 (21.98–31.04), and 20.00 (15.67–26.50). There were no significant differences in the MDS‐UPDRS part III motor examination total scores between non‐HTB and before HTB (*p* > .05; Figure 3). Compared with non‐HTB, the decrease in the MDS‐UPDRS part III motor examination total scores after HTB signified improvement in MS (*p* = .018; Table 3). Analysis of major MS (bradykinesia, rigidity, tremor, postural instability, and gait disorder) revealed a significant improvement in bradykinesia and rigidity after HTB (*p* = .043; *p* = .008; Table 3, Figure 3). Although there were no significant changes in rest tremor (items 17 and 18) and postural instability and gait disorder (items 9 and 10–13), we found a slight improvement in rest tremor amplitude (item 17; *p =* .047; Table 3) and posture (item 13; *p* = .038; Table 3) and an increase in the kinetic tremor of hands (item 16; *p =* .017; Table 3) after HTB by analyzing the 18 items in the MDS‐UPDRS part III motor examination total score. However, there was no significant difference in the modified Webster Symptoms Score between non‐HTB and after HTB (*p* > .05; Table 3).

**TABLE 3:** Effects of hyperthermic baths (HTB) on motor symptoms (MS) of Parkinson's disease (PD).

	The HTB study (*n* = 9)				
	Non *M* (P_25_–P_75_)	Before *M* (P_25_–P_75_)	After M (P_25_–P_75_)	*p* value^a^	*p* value^b^
MDS‐UPDRS part III scores	24.33 (22.33–30.83)	24.33 (21.98–31.04)	20.00 (15.67–26.50) ^**^	.953	.018^*^
Bradykinesia (items 1, 2, 14, 4–8)	10.00 (7.67−13.33)	11.33 (8.33−13.67)	10.00 (5.16−11.50) ^**^	.125	.043^*^
Rigidity (item 3)	7.33 (6.33–10.33)	7.00 (6.17–9.83)	5.33 (2.17–6.00) ^**^	.236	.008^*^
Rest tremor (items 17 and 18)	1.67 (0.50–6.50)	1.33 (0.50–6.67)	0.67 (0.17–6.50)	.516	.064
Postural instability and gait disorder (items 9, 10–13)	2.67 (2.17–3.33)	3.00 (2.33–3.67)	2.67 (2.33–3.33)	.124	.773
Speech (item 1)	0.67 (0.00–1.00)	0.67 (0.00–1.00)	0.33 (0.00–1.00)	.157	.257
Facial expression (item 2)	1.67 (1.00–2.17)	1.67 (1.00–2.17)	1.33 (0.83–2.17)	.236	.272
Finger tapping (item 4)	2.00 (1.50–2.50)	2.00 (2.00–2.50)	2.00 (1.00–2.67)	.317	.440
Hand movements (item 5)	1.67 (1.33–2.50)	1.67 (1.00–2.33)	1.00 (0.33–2.17) ^**^	.564	.017^*^
Pronation—supination movements of hands (item 6)	1.00 (0.67–1.83)	1.33 (0.83–2.00)	1.33 (0.33–1.67)	.084	.796
Toe tapping (item 7)	1.00 (0.83–2.17)	1.33 (1.00–2.17)	1.33 (0.33–1.50)	1.000	.078
Leg agility (item 8)	0.67 (0.33–1.17)	1.00 (0.50–1.17)	0.33 (0.17–0.83)	.102	.135
Arising from chair (item 9)	0.00 (0.00–0.00)	0.00 (0.00–0.17)	0.00 (0.00–0.00)	.317	1.000
Gait (item 10)	1.00 (1.00–1.33)	1.00 (1.00–1.50)	1.00 (1.00–1.50)	.157	.577
Freezing of gait (item 11)	0.00 (0.00–0.00)	0.00 (0.00–0.00)	0.00 (0.00–0.00)	1.000	.317
Postural stability (item 12)	0.00 (0.00–0.33)	0.00 (0.00–0.33)	0.00 (0.00–0.67)	1.000	.480
Posture (item 13)	1.33 (1.00–2.17)	1.33 (1.00–1.67)	1.00 (1.00–1.67)^**^	.167	.038^*^
Global spontaneity of movement (body bradykinesia) (item 14)	1.33 (1.00–1.83)	1.67 (1.00–2.00)	1.67 (1.00–2.00)^**^	.564	.046^*^
Postural tremor of hands (item 15)	0.67 (0.00–1.50)	1.00 (0.00–1.67)	0.67 (0.17–1.33)	.157	.480
Kinetic tremor of hands (item 16)	0.67 (0.00–1.33)	0.33 (0.00–1.00)	1.00 (0.17–2.33) ^**^	.083	.017^*^
Rest tremor amplitude (item 17)	1.00 (0.33–2.50)	1.00 (0.17–2.67)	0.33 (0.00–2.50)^**^	1.000	.047^*^
Constancy of rest tremor (item 18)	0.67 (0.17–4.00)	0.33 (0.17–4.00)	0.33 (0.17–4.00)	.317	.414
Modified Webster Symptoms Score	7.67 (6.83–10.17)	8.33 (7.17–10.17)	8.33 (6.83–9.33)	.429	.235

Abbreviations: *M* (P25–P75), median and interquartile range; MDS‐UPDRS part III scores, the Movement Disorder Society‐United Parkinson's Disease Rating Scale part III motor examination total scores.

^a^Efficacy analysis: Paired Wilcoxon rank test was used to adequately assess the differences between non‐HTB and before HTB.

^b^Efficacy analysis: Paired Wilcoxon rank test was used to adequately assess the differences between non‐HTB and after HTB; *p* < .05 was considered significant.

## 4 DISCUSSIONS

The results from the current study showed that seasonal variation in temperature and the water temperature of HTB may affect MS in some patients with PD. Simple HTB could provide an aquatic thermal environment, which can temporarily improve MS, especially bradykinesia, rigidity, rest tremor amplitude, and posture, so it could be recommended as physiotherapy to patients with PD who report temperature‐sensitive MS.

In phase 1 of the study, the cross‐sectional study showed that 42.86% of patients with PD felt that MS were affected by seasonal variation, and 77.01% of them felt that MS were improved after HTB. Our study showed that the MS of patients affected by seasonal variation were more susceptible to improvement after HTB. Although Postuma et al. (2005) found no relationship between season and subscores on the UPDRS, we believe that the study did not stratify patients with PD, and only a subset of patients with PD clinically had seasonal fluctuations in MS, which may reduce the sensitivity of their statistical tests for seasonality. Our clinical findings of seasonal variation in MS are consistent with the results of several studies on seasonal variation in dopamine (DA) synthesis (Eisenberg et al., 2010; Kaasinen et al., 2012; Karson et al., 1984). Compared to those in spring and summer, the synthesis and uptake of putamen DA increased in autumn and winter. The binary logistic regression analysis revealed that MS of patients with PD with higher LED tended to be influenced by seasonal variation (*p* = .095), and the incidence of patients affected by seasonal variation tended to increase from H&Y stage 1 to H&Y stage 4. It is well known that the more severe the MS of PD, the greater the LED required. In clinical practice, we also found that MS of PD were more susceptible to seasonal variation as the disease progressed. However, our data could not explain this problem statistically. The most obvious explanation is that the outpatients had relatively mild clinical conditions; thus, the number of patients in H&Y stage 5 was small. Further study could increase the number of patients in H&Y stages 5 to investigate this phenomenon.

In phase 2 of the study, eight patients showed improvement in MS, and four of the patients experienced an improvement in MS of 20% or more specifically, providing substantial evidence for the effect of seasonal variation in temperature on the MS of PD. The effects were evident in subjective feelings and measures (the MDS‐UPDRS part III motor examination total scores). Our results regarding the improvement of motor function by HTB, assessed by the MDS‐UPDRS part III motor examination total scores, were also in agreement with that of Matsumoto and Vivas (Masiero et al., 2019; Vivas et al., 2011). Moreover, the simple HTB in our study did not significantly improve gait and postural stability, but they could significantly improve bradykinesia, rigidity as well as posture. The improvement occurred immediately after the HTB, and these patients subjectively felt that the improvement of MS could be maintained for several hours. There were no statistically significant differences in the MDS‐UPDRS part III motor examination total score item 3 (resting tremor) between non‐HTB and after HTB in our study, but HTB could improve rest tremor amplitude in patients with PD. Related research also indicated that limb warming does not improve hand function (resting tremor) in patients with PD except for movements with small common objects (Cooper et al., 2000). The modified Webster Symptoms Score, only including 10 relatively general items, is not as detailed as the MDS‐UPDRS part III motor examination; hence, it might be the reason there was no significant difference in the modified Webster Symptoms Score between non‐HTB and after HTB. The participants reported that the improvement of MS after HTB in winter was more obvious than in autumn. However, the simple HTB were conducted in September and October (autumn month). Therefore, we speculate that HTB could improve MS to a greater extent in winter, especially in patients with PD who are mainly characterized by bradykinesia and rigidity. Additionally, a water temperature of 39–41°C and a bath time of 20 min are tolerable for patients. In the future, further study could determine more appropriate water temperature and bathing time to improve the MS of PD.

The maintenance of body core temperature occurs in three ways: autonomic responses, stable climate, and human behavior (Cheshire, 2016). Seasonal variation in temperature and the water temperature of HTB could influence body core temperature by changing skin temperature. Body temperature regulation is controlled by the thermoregulatory center of the body (the preoptic/anterior [PO/AH] hypothalamic region of the hypothalamus), which can modulate the balance between metabolic heat production and heat loss according to environmental conditions (Madden & Morrison, 2019; Nagashima, 2006; Tansey & Johnson, 2015). The dopaminergic pathway could regulate heat loss responses in the [PO/AH] hypothalamic region, and an increased DA release in the PO/AH will increase heat loss (Balthazar et al., 2010; Hasegawa et al., 2005; Zheng & Hasegawa, 2016; Zheng et al., 2014). A recent study indicated that thermoregulatory sub‐items of autonomic dysfunction were improved in the short‐term after subthalamic nucleus‐deep brain stimulation surgery (Zhang et al., 2022), and it improved the temperature perception of patients with PD (Witjas et al., 2007). The extent to which body temperature rises during exercise can differ according to ambient temperature (Tanaka et al., 1988; Wanner et al., 2014), and increases in DA and noradrenaline release in the preoptic/anterior hypothalamic region were more pronounced at a warm ambient temperature (Zheng et al., 2018). It might indicate that DA released in the preoptic/anterior hypothalamic region increases as ambient temperature rises. However, abnormal temperature regulation exists in patients with PD, and the pathophysiological basis of which may be that there are Lewy bodies in the hypothalamus and sympathetic ganglia (Beach et al., 2010; den & Bethlem, 1960; Langston & Forno, 1978; Wakabayashi & Takahashi, 1997). Although it is currently unclear how much DA is released in the PO/AH hypothalamic region of patients with PD at warmer temperatures, the increase in ambient temperature may also cause an increase in DA release in patients with PD, thus improving the MS of PD.

Several limitations should be borne in mind. First, the study was a single‐center study with a relatively small sample size, which may influence the reported outcome. Second, it was difficult to distinguish between the effects of “aquatic” therapy and the general thermal effects to improve motor symptoms in PD. Therefore, future studies should design a control group in which non‐bathing patients are exposed to a higher room temperature to distinguish the effects of “aquatic” therapy and the general thermal effects. Third, in order to make an attempt to explore this phenomenon, we designed these four questions based on clinical observation to form a questionnaire. Although the questionnaire illustrates certain issues, it is not rigorous enough. Lastly, the current study does not fully explain why only part of patients with PD are affected by seasonal variation, and future studies will need larger sample sizes to investigate this phenomenon.

## 5 CONCLUSION

In conclusion, our study provides further evidence that seasonal variation in temperature and water temperature of HTB may affect MS in some patients with PD. In addition, simple HTB could be recommended as physiotherapy to patients with PD who report temperature‐sensitive MS, providing an aquatic thermal environment to improve MS temporarily, especially in bradykinesia, rigidity posture, and rest tremor amplitude.

## AUTHOR CONTRIBUTIONS

**Ting Huang**: Writing—original draft; writing—review and editing; conceptualization; investigation; methodology; software; data curation; supervision. **Xi‐Xi Wang**: Conceptualization; investigation; writing—original draft; writing—review and editing; methodology; data curation; software; supervision. **Chen‐Yu Gao**: Methodology; data curation; software. **Jin‐Ying Zhao**: Methodology; software. **Rong‐Rong Pan**: Methodology; software. **Hui‐Hui Jin**: Methodology; software. **Yong Tian**: Conceptualization; resources; funding acquisition; project administration; supervision. **Ying‐Dong Zhang**: Conceptualization; resources; funding acquisition; project administration; supervision.

## CONFLICT OF INTEREST STATEMENT

The authors declare no conflicts of interest.

### PEER REVIEW

The peer review history for this article is available at https://publons.com/publon/10.1002/brb3.3369.

## Supporting information

TABLE S1. Questionnaire
TABLE S2. Demographical data and baseline characteristics of the subjects in phase 2 of the study

## Data Availability

The data that support the findings of this study are available from the corresponding author upon reasonable request.
