# Supplementary material for: Motor symptoms of Parkinson's disease are affected by temperature: A controlled pilot study
Source: Brain Behav. 2024 Jan 6;14(1):e3369. doi: 10.1002/brb3.3369 (PMC10771231; doi:10.1002/brb3.3369)
Supplement: Supplementary file 1 — TABLE S1. Questionnaire TABLE S2. Demographical data and baseline characteristics of the subjects in phase 2 of the study [file BRB3-14-e3369-s001.docx]

**TABLE S1.** Questionnaire

| Questions | 0 | 1 | 2 |
| --- | --- | --- | --- |
| Feel that MS of PD are affected by seasonal variation ^a^ |  |  |  |
| The difference in MS of PD between in winter (low temperature, less sunshine) and in summer ^b^ |  |  |  |
| Feel that MS of PD are affected by HTB ^a^ |  |  |  |
| Feel that MS of PD are improved after HTB ^c^ |  |  |  |

**Abbreviations:** MS, motor symptoms; PD, Parkinson’s disease; HTB, hyperthermic baths. a 0: no influence; 1: possible influence; 2: positive influence. b 0: no difference; 1: MS are more serious in summer; 2: MS are more serious in winter. c 0: no improvement; 1: possible improvement; 2: certain improvement.

**TABLE S2.** Demographical data and baseline characteristics of the subjects in phase 2 of the study

| Characteristics | study subjects  M (±SD) or % |
| --- | --- |
| males, n (%) | 8 (0.89) |
| Age (years) | 66.44 (7.58) |
| Age at onset (years) | 60.67 (8.53) |
| Symptoms duration (years) | 5.78 (2.82) |
| Hoehn and Yahr stage, n (%) |  |
| I | 1 (11.1) |
| II | 4 (44.4) |
| III | 4 (44.4) |
| LED | 572.22 (381.90) |
| Family history, n (%) | 2 (22.2) |
| MDS - UPDRS part I score | 9.78 (4.84) |
| MDS - UPDRS part II score | 12.89 (2.62) |
| MMSE score | 27.33 (1.94) |
| MOCA score | 26.56 (0.726) |
| Subjective improvement time (hours) | 2.50（2.11） |
| Subjective improvement rate (%) | 28.89（16.16） |

**Abbreviations:** M (±SD), mean and standard deviation; LED, Levodopa equivalent dose; MDS-UPDRS, the Movement Disorder Society-United Parkinson’s Disease Rating Scale; MMSE, Mini-Mental State Examination MOCA, Montreal Cognitive Assessment.
